# Evolutionary research on mental health in non-industrialized contexts: An introduction to the special issue

**DOI:** 10.1093/emph/eoag015

**Published:** 2026-06-25

**Authors:** Adam D Hunt, Elspeth Ready, Adrian V Jaeggi

**Affiliations:** Department of Archaeology, University of Cambridge, Cambridge, UK; Department of Human Behavior, Ecology and Culture, Max Planck Institute for Evolutionary Anthropology, Leipzig, Germany; Institute of Evolutionary Medicine, University of Zurich, Zurich, Switzerland

## THE NEED FOR EVOLUTIONARY RESEARCH ON MENTAL HEALTH

volutionary psychiatry offers novel explanations for conditions whose aetiologies remain poorly understood [1–4]. Despite many theoretical perspectives and some empirical tests in Western populations [5, 6], evolutionary psychiatrists have conducted scant research in non-industrialized contexts.

A primary question is whether conditions represent dysfunctions, or whether they were neutral or adaptive in ancestral environments [7, 8]. This can be studied by measuring fitness consequences of relevant traits in conditions such as small, interdependent social groups, daily subsistence demands, high fertility and mortality, and energetic constraints [9, 10]. A related question is whether conditions are products of evolutionary mismatch, i.e. whether they become harmful only in post-industrial contexts [11, 12]. Variation in economic systems and exposure to modern technology is uniquely informative for testing such hypotheses, and recognition of specific mismatches could help mitigate mental health problems [13]. Research of this type is pressing because of rapid economic transitions and can help local communities anticipate potential mental health consequences.

Similar prior research on mental health, e.g. on Indigenous people in North America and Australia, has often been fraught [14–16]. To instigate a more valid, concerted research effort, a workshop at the University of Zurich brought together anthropologists, psychiatrists, and cross-cultural psychologists, which led to the MAPPING Mental Health working group and the call for papers for this special issue. Although the scarcity of contributions reflects a nascent field that requires a lot of time investment (e.g. Scaff *et al*. [17]), we see this special issue as a jumping-off point for future work (see also Syme and Hagen [8]).

## THE PAPERS IN THIS SPECIAL ISSUE

An essential step in any field is to develop the right methods. Scaff *et al*. [17] make a major contribution to the problem of cross-cultural measurement of psychological traits. They begin with 12 established scales for assessing traits related to psychopathology and determine which items could be adapted for use among the Tsimane, an Indigenous population in lowland Bolivia. Out of over 400 items, about three-quarters were redundant, not culturally relevant, or were not understood in the intended way. Even the retained items required extensive modifications to be successfully used. Their paper shows that the development of adequate instruments is a major research endeavour in itself, one greatly facilitated by contributions from local experts such as experienced field assistants. Through this demanding work, Scaff and colleagues lay the foundation for future work exploring how psychological traits among Tsimane are patterned and related to various outcomes.

Cross-cultural research on mental health has shown that psychological distress is often due to a rupture in social relations [18]. Hence, treatment of psychological issues is often a social matter outside of Western medicine. Asatsa *et al*. [19] examine traditional mourning rituals among the Luhya people in Kenya. They interpret their findings from a cultural-evolutionary perspective, which highlights two key points. The first is that Luhya mourning rituals encourage resolutions to traumatic events through alignment with community norms and can provide closure for sufferers by allowing reintegration into normal social life. The second insight is that rituals are also sites of power where social norms and social hierarchies may be reinforced, which can negatively impact the well-being of vulnerable or marginalized persons (such as women). The article thus highlights a key tension in how culture influences mental health: practices that promote the well-being of some may be detrimental to others.

Bridgers and Fox [20] address the mismatch question by examining perinatal mood and anxiety disorders, which affect up to 17.7% of mothers and have become a leading cause of maternal mortality in postindustrial societies. Drawing on comparative evidence from contemporary hunter-gatherer societies, the authors identify three interrelated sources of mismatch: closer interbirth spacing, reduced allomaternal support, and lack of prior childcare experience. As allomothering was likely integral to the evolution of shorter human interbirth intervals, the absence of such support represents a particularly acute mismatch. The article highlights how evolutionary perspectives can illuminate social risk factors for maternal mental health problems, which could inform preventative interventions and screening.

Finally, Zefferman *et al*. [21] studied the hormonal underpinnings of post-traumatic stress disorder (PTSD) among Turkana pastoralists in Kenya, a population experiencing frequent combat and high mortality through livestock raids. The authors recruited 66 male Turkana who had participated in livestock raids; 21 participants reported levels of PTSD beyond standard clinical cutoffs. Using a number of preregistered statistical tests, the authors found no clear association between PTSD symptom severity and diurnal cortisol or testosterone rhythms, which contrasts with the ‘blunted’ cortisol rhythms often described for PTSD in Western samples. Similarly, their previous work had shown differences between Turkana warriors and US veterans, with the Turkana experiencing fewer depression-like symptoms despite similar levels of PTSD [22]. The study adds to growing evidence of hormonal variation and the link between mental health and hormones.

## CONCLUSION

Understanding the evolutionary roots of mental disorder and eventually improving global mental health by investigating non-industrialized communities is highly appealing, but presents several challenges. A better understanding of the variability in the causes and manifestations of mental disorders across cultures will be highly impactful. Therapies might be inspired by observed interventions or by reducing mismatches [12, 13]. We encourage researchers who can conduct this work to do so, but to be mindful of the points raised in this special issue.
